# Spectroelectrochemical Study of Carbon Monoxide and Ethanol Oxidation on Pt/C, PtSn(3:1)/C and PtSn(1:1)/C Catalysts

**DOI:** 10.3390/molecules21091225

**Published:** 2016-09-12

**Authors:** Rubén Rizo, María Jesús Lázaro, Elena Pastor, Gonzalo García

**Affiliations:** 1 Departamento de Química, Instituto de Materiales y Nanotecnología, Universidad de La Laguna, Avda. Astrofísico Francisco Sánchez s/n, La Laguna 38071, Santa Cruz de Tenerife, Spain; rjrizo57@gmail.com; 2 Instituto de Carboquímica (CSIC) Miguel Luesma Castan 4, Zaragoza 50018, Spain; mlazaro@icb.csic.es

**Keywords:** ethanol electrooxidation, Pt-Sn electrocatalysts, DEMS, FTIRS, direct ethanol fuel cell

## Abstract

PtSn-based catalysts are one of the most active materials toward that contribute ethanol oxidation reaction (EOR). In order to gain a better understanding of the Sn influence on the carbon monoxide (principal catalyst poison) and ethanol oxidation reactions in acidic media, a systematic spectroelectrochemical study was carried out. With this end, carbon-supported PtSn_x_ (x = 0, 1/3 and 1) materials were synthesized and employed as anodic catalysts for both reactions. In situ Fourier transform infrared spectroscopy (FTIRS) and differential electrochemical mass spectrometry (DEMS) indicate that Sn diminishes the amount of bridge bonded CO (CO_B_) and greatly improves the CO tolerance of Pt-based catalysts. Regarding the effect of Sn loading on the EOR, it enhances the catalytic activity and decreases the onset potential. FTIRS and DEMS analysis indicate that the C-C bond scission occurs at low overpotentials and at the same potential values regardless of the Sn loading, although the amount of C-C bond breaking decreases with the rise of Sn in the catalytic material. Therefore, the elevated catalytic activity toward the EOR at PtSn-based electrodes is mainly associated with the improved CO tolerance and the incomplete oxidation of ethanol to form acetic acid and acetaldehyde species, causing the formation of a higher amount of both C2 products with the rise of Sn loading.

## 1. Introduction

Fuel cells are widely recognized as an important topic of research for being an alternative power supply which allows the reduction of greenhouse gas emissions [1,2,3]. Since ethanol is the major renewable bio-fuel and less toxic than other alcohols, it is a promising power source for Direct Alcohol Fuel Cells (DAFCs) [1,2,3,4,5]. The full oxidation of ethanol to carbon dioxide has a favorable thermodynamic potential of 0.08 V (vs. RHE), although the efficiency of direct ethanol fuel cells is drastically limited by the formation of acetaldehyde, acetic acid and strongly adsorbed intermediates such as carbon monoxide (CO_ad_) [1,2,3,4,5,6,7,8,9,10,11,12,13,14,15,16,17,18,19]. The ethanol oxidation reaction (EOR) at Pt-based electrodes is complicated and still not fully clear, although recently we reported new insights in its reaction mechanism [6,7]. At low overpotentials, the initial steps involve the dissociative adsorption of ethanol to produce adsorbed intermediate species. In this context, ethanol possesses two reactive sites (OH group and the α-carbon atom) that may interact with Pt surface sites, and therefore two possible adsorbate species can be considered (Equations (2) and (3)):

Pt + CH_3_CH_2_OH ⇌ Pt(CH_3_CH_2_OH) (1)

Pt(CH_3_CH_2_OH) ⟶ Pt-OCH_2_CH_3_ + H^+^ + e^−^ (2)

Pt(CH_3_CH_2_OH) ⟶ Pt-CHOHCH_3_ + H^+^ + e^−^ (3)

At E < 0.4 V (in the hydrogen adsorption/desorption region), alcohol intermediate species reacts mainly with adsorbed hydrogen (H_ad_) on defect sites with (100) orientation to produce methane and adsorbed CO. The last is supported by the relatively stability of the ethoxi species and because the alcohol intermediate species appears to be favored in acidic media:

Pt_100_(H) + Pt-CHOHCH_3_ ⟶ Pt-CO + CH_4_ + 2H^+^ + 2e^−^ (4)

At E > 0.4 V (after the hydrogen adsorption/desorption region), further deprotonation of the adsorbate formed in Equation (3) produces acetaldehyde:

Pt-CHOHCH_3_ ⟶ Pt(HCOCH_3_) + H^+^ + e^−^ (5)

Pt(HCOCH_3_) ⇌ Pt + HCOCH_3_ (6)

In addition to acetaldehyde, further deprotonation of the intermediate formed in Equation (3) may produce adsorbed CH_3_ and CO species:

Pt-CHOHCH_3_ + Pt ⟶ Pt_2_-COHCH_3_ + H^+^ + e^−^ (7)

Pt_2_-COHCH_3_ ⟶ Pt-CH_3_ + Pt-CO + H^+^ + e^−^ (8)

Then, at more positive potentials (E > 0.6 V for Pt/C), in which the water dissociation reaction occurs (Equation (9)), the following reactions may happen:

Pt + H_2_O ⇌ Pt-OH + H^+^ + e^−^ (9)

Pt-CO + Pt-OH ⟶ 2Pt + CO_2_ + H^+^ + e^−^ (10)

Pt(HCOCH_3_) + Pt-OH ⟶ 2Pt + CH_3_COOH + H^+^ + e^−^ (11)

Pt-CH_3_ + 2Pt-OH ⟶ 3Pt + CO_2_ + 5H^+^ + 5e^−^ (12)

It is remarkable that reaction (12) does not represent the elementary steps of the oxidation of adsorbed CH_3_ species, which probably involves a CO-like intermediate.

In order to enhance the performance of DAFC, one of the principal strategies involves the development of new materials to enhance the water dissociation reaction (Equation (9)) and therefore improve the oxidation reaction of strongly adsorbed species (Equations (10) and (12)), which are the main catalyst poisons. In this regard, PtSn-based catalysts are the most employed materials for the ethanol oxidation reaction (EOR) in acidic media. Sn seems to provide oxygenated species (Equation (9)) at more negative potentials than Pt and therefore the CO tolerance increases at PtSn-based electrodes. In this sense, previous studies for ethanol oxidation on PtSn-based catalysts reported lower overpotentials and higher current density during the EOR in comparison to Pt electrodes [9,10,11,12,13,14,15,16,17,18,19,20,21,22,23,24,25,26,27,28,29,30,31,32,33]. In situ Fourier transform infrared spectroscopy (FTIR) was employed by Lamy et al. [33] to study the EOR at PtSn-based electrodes. They suggested that the presence of Sn on Pt electrodes increases the dissociative adsorption of ethanol (and therefore favours the C-C bond breaking) at low overpotentials and the production of acetic acid and acetaldehyde at high overpotentials. Differential electrochemical mass spectrometry (DEMS) was employed by Behm et al. [29] to follow the volatile and gaseous products and intermediates generated during the EOR at carbon-supported PtSn and PtRu. They found that the addition of Sn or Ru into the Pt-based catalyst decreases the overpotentials for the EOR and better catalytic activity at carbon-supported Pt_3_Sn catalyst was observed. Nevertheless, they reported that the insertion of Sn or Ru does not improve the ethanol conversion efficiency to CO_2_.

In order to gain a deeper understanding of the operating mechanism for the EOR at PtSn-based catalysts, the present work employ carbon-supported PtSn with different Pt:Sn atomic ratios (3:1 and 1:1) and the results are compared with those achieved at carbon supported Pt. The EOR in acidic media was studied by conventional (cyclic voltammetry and chronoamperometry) and non-conventional spectroelectrochemical (DEMS and FTIRS) techniques.

## 2. Results and Discussion

### 2.1. Physicochemical Characterization

The physicochemical parameters of all catalysts were studied by TEM, XRD, EDX and XPS techniques and reported in our previous work [24]. Main important outcomes indicate: (i) similar metal distribution onto the carbon support (TEM); (ii) similar particle sizes (3–5 nm, XRD and TEM); (iii) similar metal content and Pt:Sn bulk atomic ratios to the nominal ones (EDX); (iv) similar Pt:Sn surface atomic ratios and Pt:Sn bulk atomic ratios (XPS and EDX), which suggest negligible metal segregation to the surface; (v) similar chemical state of Pt and Sn species into the surface (XPS) (vi) lattice parameter values, degree of alloying between Pt and Sn and the relative amount of crystalline SnO_2_ phases increase in the order Pt < Pt–Sn 3:1 < Pt–Sn 1:1 (XRD).

### 2.2. Adsorbed CO ELectrooxidation (CO Stripping)

The activities of the catalysts toward carbon monoxide electrooxidation provide insight about their tolerance toward CO poisoning, which is one of the most important issues concerning the alcohol electrooxidation in direct alcohol fuel cell anodes, as adsorbed CO (CO_ad_) is the principal catalyst poison produced during alcohol oxidation [2,34]. Therefore, CO_ad_ monolayer oxidation measurements (“CO stripping experiments”) were performed at 0.005 V·s^−1^ in sulphuric acid solution at room temperature. The anodic charge obtained from the CO stripping measurements was used to achieve the electroactive surface area (ECSA).

**Figure 1:**
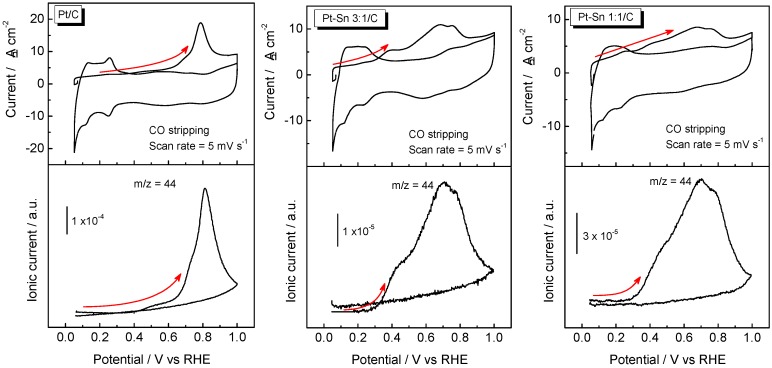
Cyclic voltammograms (CV) and mass spectrometric CV (MSCV) for CO stripping. Faradaic current (**top panel**) and ionic current for *m*/*z* = 44 signal (**bottom panel**) registered during a CO_ad_ monolayer electrooxidation on Pt/C, Pt–Sn 3:1/C and Pt–Sn 1:1/C in 0.5 M H_2_SO_4_ at room temperature. v = 0.005 V·s^−1^.

Cyclic voltammograms (CVs) and mass spectrometric cyclic voltammograms (MSCVs) for the oxidative desorption of a CO monolayer previously adsorbed at 0.07 V vs. RHE were recorded simultaneously. Figure 1 shows the CVs (top panel) and the corresponding MSCVs (bottom panel) for the *m*/*z* = 44 (CO_2_^+^) signal for all catalysts in acidic media recorded at 0.005 V·s^−1^. It is observed that faradaic and ionic currents rise and develop a peak centered at 0.79 V for Pt/C electrode. On the other hand, a broad anodic current peaking at ca. 0.38 V, 0.68 and 0.76 V was developed for both PtSn-based materials. The latter is confirmed by the MSCVs, which indicate that the CO oxidation reaction takes place in a broad potential region. Therefore, it seems that the CO surface diffusion toward the most Pt active site is more impeded at PtSn-based catalysts than at Pt/C electrode [2,5,10]. Another important parameter to study the CO tolerance is the onset potential for the CO oxidation reaction. In this regard, it is clearly observed that the insertion of Sn into the catalyst strongly enhances the CO tolerance since the onset potential is about 0.25 V more negative at PtSn-based electrodes than at Pt/C catalyst. Interestingly, both PtSn materials develop similar onset potential, suggesting that the CO tolerance is not strongly influenced by the amount of Sn. In order to gain a better understanding of the CO oxidation reaction at PtSn-based electrodes, FTIRS experiments were carried out.

**Figure 2:**
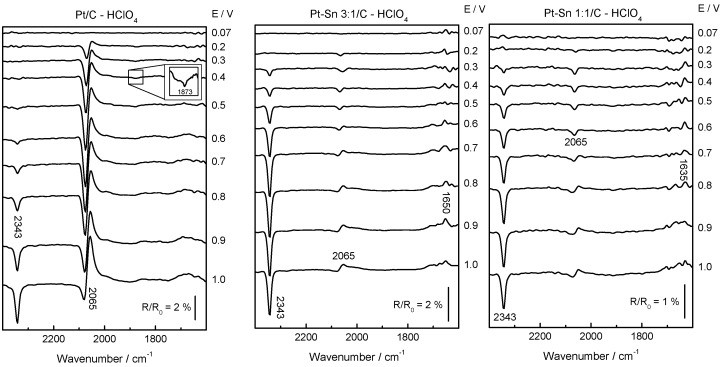
In situ Fourier transform infrared spectroscopy (FTIR) spectra recorded during a CO_ad_ monolayer electrooxidation on Pt/C, Pt–Sn 3:1/C and Pt–Sn 1:1/C catalysts in 0.1 M HClO_4_ at room temperature. E_ad_ = R_0_ = 0.07 V.

In situ FTIRS was employed to investigate the CO_ad_ oxidation process on Pt/C, Pt–Sn 3:1/C and Pt–Sn 1:1/C catalysts in perchloric acid medium to avoid the IR absorption by sulfate species at wavenumbers lower than 1400 cm^−1^. Therefore, a series of spectra acquired during the oxidation of a monolayer CO, with a reference spectrum obtained at 0.07 V, is given in Figure 2. The negative band at 2343 cm^−1^ corresponds to the production of CO_2_ and it is apparent at E ≥ 0.50 V for Pt/C and at E ≥ 0.3 V for PtSn-based materials. The last is in complete agreement with previous DEMS results. Another signal is observed that first appears as bipolar at ca. 2080–2030 cm^−1^ and turns positive at higher potentials. The bipolar feature is related to an adsorbate that is still present at the surface and suffers an important wavenumber shift as a consequence of increasing the potential (stark effect) [34]. In this case, this band is well known and is related to linear adsorbed CO (CO_L_). Also, a small band is apparent at 1873 cm^−1^ that is associated with bridge bonded CO (CO_B_). The intensity of the last band decreases with the Sn loading in the material, being absent at the Pt–Sn 1:1/C catalyst.

Therefore, both in situ techniques (DEMS and FTIRS) reveal an enhancement of the CO tolerance by Sn, and the bifunctional mechanism [1,2,3,4,5,9,10,11,12,13,14,15,16,35,36] appears to be mainly responsible since physicochemical analysis indicate negligible electronic effects by Sn addition [24].

### 2.3. Ethanol Electrooxidation

**Figure 3:**
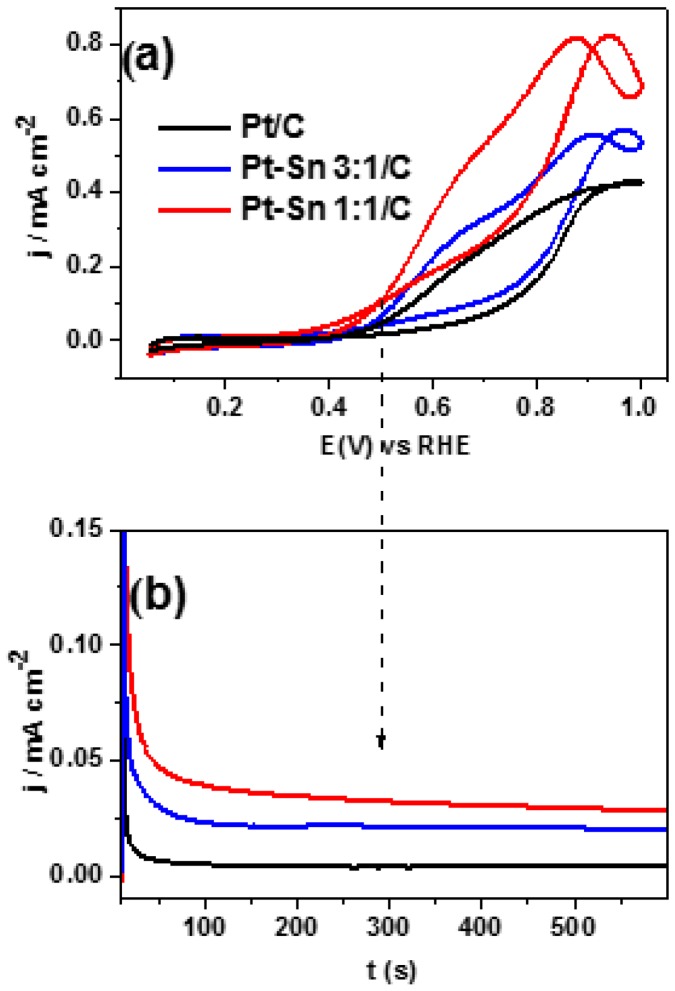
Ethanol electrooxidation on Pt/C, Pt–Sn 3:1/C and Pt–Sn 1:1/C catalysts in 1 M CH_3_CH_2_OH + 0.5 M H_2_SO_4_ at room temperature. (**a**) CVs recorded at 0.02 V·s^−1^; (**b**) current-transients recorded at 0.5 V.

Ethanol electrooxidation reaction (EOR) was studied on Pt/C, Pt–Sn 3:1/C and Pt–Sn 1:1/C catalysts by cyclic voltammetry and chronoamperometry at room temperature. The working electrode was introduced at controlled potential (0.05 V vs. RHE) where the oxidation of the ethanol molecule is negligible and afterwards CVs were performed between 0.05 and 1 V vs. RHE at 0.02 V·s^−1^ (Figure 3a) or a potentiostatic pulse at 0.5 V was applied during 10 min (Figure 3b). As was described before, the faradaic currents were normalized by the ECSA calculated from CO stripping experiments. Thus, Figure 3a shows the third CVs (subsequent CVs reveal similar and stable profiles) for the three catalysts. It is observed an increment of the faradaic current and a diminution of the onset potential with the rise of the Sn loading. In agreement with CVs results, current transients recorded at 0.5 V (Figure 3b) reveal an enhancement of the catalytic activity toward the ethanol oxidation reaction (EOR) with the rise of the Sn loading. Therefore, the catalytic activity toward the EOR increases in a subsequent way: Pt/C < Pt–Sn 3:1/C < Pt–Sn 1:1/C. These results are in agreement with those reported by Tsiakaras et al. [12], in which Pt-Sn catalysts with metallic ratios ranging from Pt_1_Sn_1_ to Pt_4_Sn_1_ were employed. They found that the maximum power density obtained in a direct ethanol fuel cell (DEFC) exhibits a “volcano-type” behavior with the Sn content in the catalyst. It was reported that the optimal Sn loading is about 30%–40% for intermediate working temperatures (60 < T < 90 °C) [13], and slightly increases (50%) for room working temperature [14,15]. In this regard, we recently reported that catalytic materials containing 75% of Sn show similar performances toward the EOR to those including 50% of Sn loading under steady-state conditions [24]. However, the results belonging to the Pt_1_Sn_3_ catalyst are not included in the current work due to the low intensity of the IR spectra.

**Figure 4:**
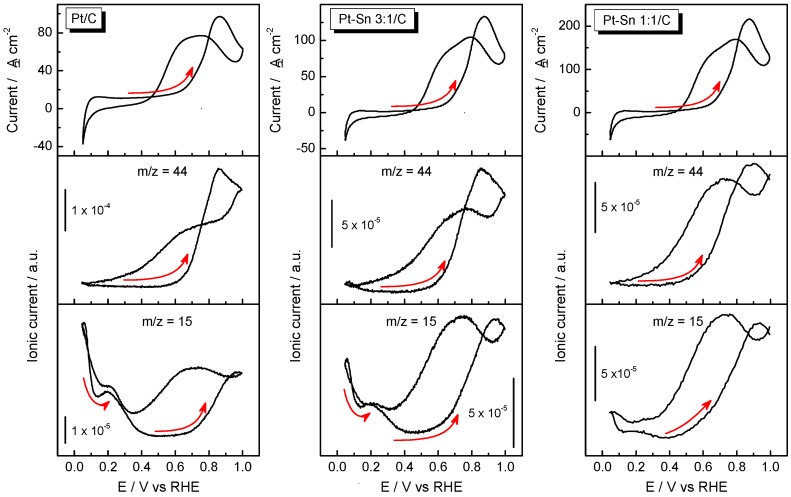
Ethanol electrooxidation on Pt/C, Pt–Sn 3:1/C and Pt–Sn 1:1/C catalysts in 1 M CH_3_CH_2_OH + 0.5 M H_2_SO_4_ recorded at room temperature and 0.005 V·s^−1^. CVs (**top panel**) and MCVs for *m*/*z* = 44 (**middle panel**) and *m*/*z* = 15 (**bottom panel**).

The formation of gaseous and volatile intermediates and products during the EOR on Pt/C, Pt–Sn 3:1/C and Pt–Sn 1:1/C catalysts was followed by DEMS. Figure 4 shows the third CVs and the corresponding MSCVs for the *m*/*z* = 15 (CH_3_^+^) and *m*/*z* = 44 (CO_2_^+^ and CH_3_CHO^+^) signals during the electrooxidation of ethanol at Pt/C, Pt–Sn 3:1/C and Pt–Sn 1:1/C electrodes in acidic media recorded at 0.005 V·s^−1^. The signals for *m*/*z* = 15 and 44 can be related to the production of two species. Indeed, the *m*/*z* signal 15 is associated with methane and acetaldehyde production (CH_3_^+^ fragment from both compounds) at low (E < 0.5 V in the forward scan and E < 0.3 V in the negative sweep) and high (E > 0.5 V in the forward scan and E > 0.3 V in the negative sweep) potentials, respectively [6,7]. The signal for *m*/*z* = 15 could be associated with the production of acetaldehyde in the whole potential range. However, a detailed comparison of the MSCVs for *m*/*z* = 15 and 44 (the latter related to acetaldehyde and/or carbon dioxide formation at E > 0.5 V), shows that both MSCVs are similar for E > 0.5 V but at E < 0.5 V the features in the MSCV for *m*/*z* = 15 are not present for *m*/*z* = 44. Therefore, in this potential range the signal for *m*/*z* = 15 has to be related to the formation of methane (Equation (4)). Methane formation is of main relevance because only can take place after C-C cleavage. In this regard, our group recently reported for the first time the production of methane at Pt/C and mesoporous Pt during the anodic scan at E > 0.1 V [6,7]. A close inspection of Figure 4 reveals an increment of acetaldehyde and/or carbon dioxide with the rise of Sn loading in the catalysts (Equations (6) and (12)), meanwhile the opposite happens with the methane production. Additionally, it is perceived that the onset potential for the *m*/*z* = 44 shifts toward more negative potentials as the amount of Sn increases.

All these results suggest that C-C bond breaking is disfavored and consequently by-side products (acetaldehyde and acetic acid) increase with the amount of Sn into the catalytic material. Indeed, methane production decreases and the *m*/*z* = 44 signal increases with the rise of the Sn loading. As was described above, the *m*/*z* = 44 signal can be related to acetaldehyde and/or carbon dioxide production. So, in order to discern between both products and gain a better understanding of the EOR, FTIRS experiments were conducted.

**Figure 5:**
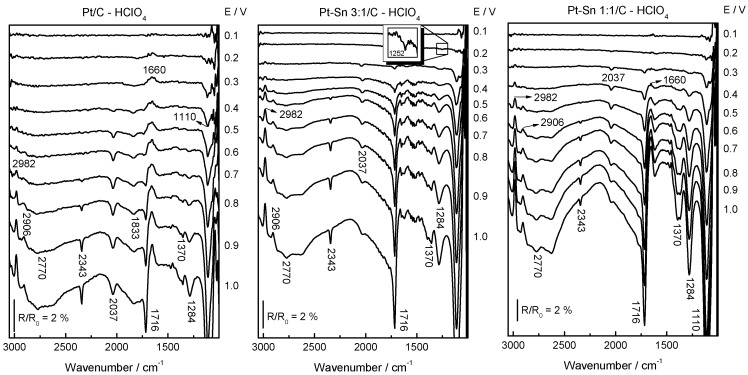
In situ FTIR spectra recorded during the ethanol electrooxidation on Pt/C, Pt–Sn 3:1/C and Pt–Sn 1:1/C catalysts in 1 M CH_3_CH_2_OH + 0.1 M HClO_4_. R_i_ = R_0_ = 0.05 V.

Figure 5 shows sequences of in situ FTIR spectra recorded during ethanol electrooxidation on Pt/C, Pt–Sn 3:1/C and Pt–Sn 1:1/C in perchloric acid media to avoid the IR absorption by sulfate species, while varying the electrode potential stepwise from 0.05 to 1.0 V (R_0_ = 0.05 V). From these spectra, similar bands appear for all catalysts that develop with different intensity and potential dependence according to the Sn loading in the catalytic material. Pt/C catalyst develops two positive bands at ca. 2982 and 2906 cm^−1^, as well as eight negative-going contributions are apparent around 2770, 2343, 2037, 1716, 1650, 1370, 1284, 1110 cm^−1^. We consider now the assignment of these bands. The small band around 1650 cm^−1^ is due to the O–H bending mode of water, which may disturb the spectral region between 1700 and 1400 cm^−1^, meanwhile the signal at ca. 1110 cm^−1^ is related to the electrolyte (perchlorate ions) that hinders signals close to this spectral region. The positive going (loss) bands at 2982 and 2906 cm^−1^ are related to the consumption of ethanol (asymmetric stretching vibrations of CH_3_ and CH_2_, respectively). The negative going bands (gain) at 2343 cm^−1^ is due to the asymmetric stretching vibration of CO_2_ and the negative contribution at ca. 2037 cm^−1^ is related to linearly adsorbed CO on Pt (CO_L_). The band at around 1716 cm^−1^ (C=O) is assigned to the *v*(C-O) stretching mode of the carbonyl groups in both acetaldehyde and/or acetic acid, but actually it is very difficult to differentiate a carbonyl from an acid or aldehyde group because the C=O band for both groups are separated by around 5 cm^−1^ in the spectra [4,17,37,38,39,40]. Nevertheless, acetic acid formation can be discerned from the bands at 2770 and 1284 cm^−1^ that are associated with the O-H stretching and O-H deformation vibrations in acetic acid. The broad band at ca. 1370 cm^−1^ is attributed to acetic acid (C-O stretching) and acetaldehyde (CH_3_ symmetric deformation and C-H wagging vibrations) [38]. Interestingly, Pt/C is the only catalyst that develops a negative contribution about 1833 cm^−1^ that is associated with bridge bonded CO (CO_B_). Additionally, PtSn-based electrodes develop at E < 0.4 V a small but visible negative band at 1252 cm^−1^. According with data in the literature, a band located approximately at this wavenumber is expected for an adsorbed tertiary alcohol (C-OH stretch), which may be in the current work either as adsorbed COH or COHCH_3_ species [34,39,40].

At E ≥ 0.2 V, adsorbed CO (CO_B_ for Pt/C and CO_L_ for Pt-Sn based electrodes) is produced (Equation (4)) that is in agreement with DEMS results in which methane was elucidated during the forward scan in the same potential range (*m*/*z* = 15 in Figure 4). Therefore, it can be stated that C-C cleavage occurs at Pt-based electrodes at low overpotentials in acidic media [6,7]. Moreover, the presence of Sn diminishes the production of methane and CO_ad_ species and therefore it seems to hinder C-C scission.

Carbon dioxide formation (Equations (10) and (12)) is apparent at E ≥ 0.7 for Pt/C and at E ≥ 0.5 V for PtSn-based electrodes. Thus, Sn improves the fuel conversion efficiency at E < 0.5 V mainly by the bifunctional effect as was described before during the CO stripping experiments and confirmed by the physicochemical analysis in which electronic effects were not discerned [24]. In this regard, Sn provides adsorbed oxygenated species at more negative potentials than Pt (Equation (9)) and therefore the CO tolerance of the material is enhanced (Equations (10) and (12)). Nevertheless, the production of carbon dioxide decreases with the rise of Sn loading, which may be ascribed to a diminution of the C-C cleavage at PtSn-based materials. The latter may be occasioned by a reduction of the active phase (i.e., Pt) by the second element.

Interestingly, the band at ca. 2770 cm^−1^, which is associated only to acetic acid (Equation (11)), increases at E ≥ 0.7 and E ≥ 0.5 V for Pt/C and PtSn-based electrodes, respectively, i.e., the same potential values for carbon dioxide formation (Equations (10) and (12)). Indeed, the water dissociation reaction (OH_ad_ formation, Equation (9)), which is enhanced by Sn species, is necessary for the production of both species. In this context, the band at ca. 1284 cm^−1^ must be used with great caution since Nafion (a catalyst ink component) absorbs IR radiation in this spectral region [41].

The onset potential for the acetaldehyde formation (Equations (5) and (6)) is clearly discerned from the negative contribution at ca. 1716 cm^−1^. The last increases at higher potentials than 0.3 and 0.5 V for PtSn-based and Pt/C catalysts, respectively. Interestingly, these potential values are in agreement with those developed in the CVs (Figure 3a). In addition, it is observed that the intensity of this band increases with the rise of Sn loading into the catalytic material. It must be taken into account that this band belongs only to acetaldehyde at potentials lower than 0.5 and 0.7 V for PtSn-based and Pt/C catalysts, respectively. At higher potentials, acetic acid also absorbs IR radiation at this wavenumber.

## 3. Materials and Methods

### 3.1. Catalysts Preparation and Physicochemical Characterization

The preparation and physicochemical characterization of the catalysts have been already described in our previous work [24]. Briefly, Pt and PtSn catalysts supported on commercial carbon Vulcan XC-72R (Cabot Co., Boston, MA, USA) were prepared by the formic acid method (FAM) at 80 °C [42]. Appropriate amounts of metal precursors (H_2_PtCl_6_ and SnSO_4_, Sigma-Aldrich, St. Louis, MO, USA) were slowly added to the previous carbon dispersion to obtain a metal loading of 20 wt %. Transmission electron microscopy (TEM, JEOL-2000 FX II microscope, Tokyo, Japan), X-ray diffraction (XRD, PANalytical X’Pert Pro X-ray diffractometer, Eindhoven, The Netherlands), energy-dispersive X-ray spectroscopy (EDX, coupled to the scanning electron microscope Jeol JSM 6300) and X-ray photoelectron spectroscopy (XPS, Thermo-Scientific equipment, Waltham, MA, USA) were employed for the physicochemical characterization of catalysts. Main results are described below during the results and discussion section.

### 3.2. Electrochemical Characterization

The catalyst ink was prepared by mixing 2 mg of the catalyst in 0.5 mL of ultrapure water (Millipore, Darmstadt, Germany, 18.2 MΩ·cm^−1^ of resistivity) and 15 μL of Nafion (5 wt %, Sigma-Aldrich). This mixture was dispersed in an ultrasonic bath and an aliquot of the suspension was pipetted on the top of the working electrode, consisting of a glassy carbon disk (7 mm), and dried at ambient temperature under N_2_ atmosphere. After preparation, the electrode was immersed into the electrochemical cell at controlled potential of 0.05 V.

A thermostated three electrodes electrochemical cell was used to perform all the experiments. This cell allows solution exchange under working electrode potential control. A carbon rod was used as counter electrode and a reversible hydrogen electrode (RHE) in the electrolyte as reference electrode. All potentials in this work are given against the RHE. Electrochemical measurements were performed with a PC Autolab potentiostat-galvanostat PGSTAT30.

Experiments were carried out in 0.5 M aqueous sulphuric solutions prepared from high purity reagents (Merck p.a., Kenilworth, NJ, USA) and ultra-pure water (Millipore MilliQ gradient A10 system, 18.2 MΩ cm, 2 ppb total organic carbon). Argon (N50) was used to deoxygenate all solutions and CO (N47) to dose CO. CO stripping experiments were obtained after bubbling CO through the cell for 15 min while keeping the working electrode at 0.07 V, followed by argon purging and electrolyte exchange to remove the excess of CO. CO stripping voltammograms were recorded, by first scanning negatively until 0.05 V so that entire hydrogen region was explored, and then scanning positively up to 1.0 V.

The charge involved in the CO oxidation peak was used to determine the electroactive surface area (ECSA), assuming a charge of 388 µC·cm^−2^ involved in the oxidation of 0.93 monolayer of linearly adsorbed CO. Current densities given in the present paper were calculated with the previously achieved ECSA.

Potentiodynamic and potentiostatic experiences of ethanol (Merck p.a.) oxidation were performed with 1 M alcohol concentration. First the working electrode was fixed to 0.05 V and later the alcohol solution was introduced into the electrochemical cell. Next, cyclic voltammograms (CVs) or current transients (CTs) were obtained by sweeping/stepping the potential from 0.05 V to the final oxidation potential.

### 3.3. EC-MS Set-Up

Gaseous and volatile species produced on the electroactive surface were continuously detected by a new electrochemical mass spectrometry (EC-MS) configuration recently reported [6,7]. Briefly, the analysis system is a commercial mass spectrometer (Omnistar™, Pfeiffer, Asslar, Germany) with a PTFE capillary (Supelco) as inlet. The electrochemical mass electrode (EC-ME) consists of a PTFE capillary fixed in a carbon disk with a hole in the middle and a small porous PTFE membrane (Gore-Tex) located onto the tip. Then, 10 μL of the catalytic ink was dried onto the PTFE membrane of EC-ME, so only very small amounts of catalysts are needed for the studies. With this EC-MS set-up, a meniscus configuration can be adopted in a conventional electrochemical cell. Thus, mass spectrometry cyclic voltammograms (MSCVs) can be recorded simultaneously with the corresponding cyclic voltammograms (CVs).

### 3.4. In-Situ FTIRS

FTIRS experiments were performed with a Bruker Vector 22 spectrometer equipped with a mercury cadmium telluride detector. A small glass flow cell with a 60° CaF_2_ prism at its bottom was used. The cell and experimental arrangements have been described in detail elsewhere [35,43]. FTIR spectra were acquired from the average of 128 scans, obtained with 8 cm^−1^ resolution at selected potentials, by applying 0.05 V single potential steps from a reference potential, in the positive going direction. The reflectance ratio R/R_0_ was calculated, where R and R_0_ are the reflectances measured at the sample and the reference potential, respectively. In this way, positive and negative bands represent the loss and gain of species at the sampling potential, respectively.

The working electrodes consisted of a certain amount of the metal/C catalysts deposited as a thin layer over a polycrystalline gold disk. The geometric area of the disk was 0.785 cm^2^. An aqueous suspension of 4.0 mg·mL^−1^ of the metal/C catalyst was prepared by ultrasonically dispersing it in 15 mL of Nafion (5 wt %, Aldrich) and pure water (Millipore). An aliquot (20 mL) of the dispersed suspension was pipetted on the top of the gold disk and dried at ambient temperature. In order to avoid the absorption by sulfate species, experiments were carried out in 0.5 M perchloric solutions prepared from high purity reagents (Merck p.a.) and ultra-pure water.

## 5. Conclusions

Carbon monoxide and ethanol oxidation reactions at carbon-supported Pt, Pt–Sn 3:1 and Pt–Sn 1:1 in acidic media were scrutinized by conventional (cyclic voltammetry and chronoamperometry) and non-conventional spectroelectrochemical techniques (FTIRS and DEMS).

CO tolerance increases with the addition of Sn into the catalytic material, which is mainly associated with the bifunctional effect. FTIRS analysis indicates a diminution of the bridge bonded CO (CO_B_) at Pt with the Sn loading in the catalytic material.

The catalytic activity toward the ethanol oxidation reaction (EOR) was found to increase in the following order: Pt/C < Pt–Sn 3:1/C < Pt–Sn 1:1/C. The opposite trend was found for the onset potential of the EOR. In this regard, FTIRS and DEMS analysis showed that the C-C scission occurs at low overpotentials and at the same values independently of the Sn loading. However, the amount of C-C bond breaking decreases with the rise of Sn in the catalyst, which is attributed to the dilution of the active phase. Additionally, both spectroelectrochemical techniques indicate that the catalytic activity and the onset potential for the EOR are improved by the promotional effect of Sn on bimetallic PtSn/C catalysts for the water oxidation reaction. The last provides oxygenated species (OH_ad_) at low overpotentials (about 0.5 V) to liberate the active phase from the main catalyst poison (CO_ad_) and to produce acetic acid. Indeed, it was detected that acetic acid and acetaldehyde increases meanwhile carbon dioxide decreases with the Sn loading in the catalyst, indicating that the enhanced catalytic activity toward the EOR is mainly due to the incomplete EOR.
